# Social and Poor-Law Problems

**Published:** 1908-10-03

**Authors:** 


### SOCIAL AND POOR-LAW PROBLEMS.

### NURSERY SCHOOLS.

The "nursery schools" which are proposed by the committee which has been investigating the school attendance of children under five years of age will, if established, form an acceptable compromise between the advice of the hygienists, who have for so long protested against the attempt to inflict school teaching and school discipline upon infants, and the parents, who, very comprehensibly, wish to have them out of their way. They may be called schools, but in effect they will be nurseries, which will give the offspring of the poor advantages similar to those enjoyed by the children of the more fortunate classes. A schoolmaster of the olden type would wax very indignant at the idea of school furniture which included "net-beds, or other suitable and sanitary provision for sleeping; large sand-troughs on wheels, for planting seeds and flowers, making sand-pies, shell impressions, etc., with a supply of small spades and buckets; pets, such as goldfish and birds; and swings, a rocking-horse, reins, balls, dolls, doll's-house, Noah's ark, and models of animals." But there is no doubt that the children will approve. There is no doubt either that the health of the children, which is the main consideration at their age, will be better looked after in the nursery school than it would be at home, or in their previous playground of the street; nor will their mental development suffer from their faculties not being consciously exercised until they are over five years of age. The captious may indeed object to the use of the word "school" in connection with a place in whose rules it may be laid down that "freedom of movement, constant change of occupation, frequent visits to the playground, and opportunities for sleep are essential," but the name matters little, and the mere fact of the association of thirty children—to which size classes are to be limited—of nearly the same age, under the care of a teacher who has a "motherly instinct" and a bright personality, will have a disciplinary effect which will tell for good afterwards when they go under more formal education. From the point of view of the children the scheme is admirable, and doubtless it will prove equally satisfactory from that of hygiene and education.

